# Exogenous Insulin Antibody Syndrome: An Overlooked Cause of Severe Hypoglycemia and Insulin Resistance in a Patient With Type 2 Diabetes Treated With Insulin

**DOI:** 10.1002/jgf2.70085

**Published:** 2025-11-26

**Authors:** Kazuki Miyaue, Hiroki Isono

**Affiliations:** ^1^ Department of General Medicine HITO Medical Center Shikokuchuo Ehime Japan

## Abstract

We report an 80‐year‐old man with type 2 diabetes who presented with life‐threatening hypoglycemia (36 mg/dL) and coma, requiring high‐dose insulin (74 units/day). Exogenous insulin antibody syndrome (EIAS) was suspected due to his glycemic instability and high insulin requirements. Subsequent testing confirmed elevated insulin antibodies (8.7 U/mL). Switching insulin analogues (to 32 units/day) promptly achieved stable glycemic control. This case highlights that EIAS, an under‐recognized cause of unpredictable glycemia in insulin‐treated diabetes, should be considered in patients with severe hypoglycemia and high insulin requirements. Modifying the insulin regimen is key for diagnosis and treatment.

## 1 Background

Severe hypoglycemia is a critical complication in insulin‐treated diabetes, often attributed to factors like excessive insulin doses, reduced carbohydrate intake, or increased insulin sensitivity [1]. However, a distinct and less frequently identified cause involves an immunological response known as exogenous insulin antibody syndrome (EIAS), where antibodies form against the administered insulin, potentially leading to significant glycemic variability [2]. Despite its potential clinical impact, EIAS remains an under‐recognized condition, often absent from prominent clinical guidelines detailing the evaluation of hypoglycemia in individuals with diabetes [1, 3].

Here, we present a case of an elderly man with long‐standing type 2 diabetes who developed life‐threatening hypoglycemia while receiving high doses of insulin. Subsequent investigation revealed EIAS as the underlying cause. This case illustrates the critical need to consider EIAS in the differential diagnosis for patients presenting with severe glycemic instability and insulin resistance and demonstrates the effectiveness of modifying the insulin regimen for both diagnosis and management.

## 2 Case Presentation

An 80‐year‐old man presented to the emergency department with unresponsiveness and depressed consciousness. The patient had a long‐standing history of type 2 diabetes mellitus and had been receiving insulin therapy long‐term. His diabetes treatment regimen, managed at another facility, consisted of empagliflozin 25 mg daily, insulin lispro (16 units morning, 4 units noon, 10 units evening), and insulin glargine (44 units morning), totaling 74 units of insulin per day. His HbA1c had generally been maintained around 7%. His recent history was notable for worsening glycemic instability, including frequent blood glucose readings exceeding 300 mg/dL and a hypoglycemic episode requiring hospitalization several months prior. He had spent the day before admission as usual. Around midnight on the day of admission, his wife noticed he was unresponsive and called for emergency services, leading to his transport to our hospital.

On presentation to the emergency department, the patient was hypertensive with a blood pressure of 185/100 mmHg and tachycardic with a heart rate of 102 beats per minute. His respiratory rate was 18 breaths per minute with snoring‐like respirations, and his oxygen saturation was 93% while receiving 2 L of oxygen via nasal cannula. Body temperature was 35.9°C. His height was 163 cm, weight was 70 kg. Neurologically, he was comatose. The eyelash reflex was absent, but pupillary light reflexes were present bilaterally. Slow, horizontal roving eye movements were observed. Muscle tone was decreased in all four limbs.

Initial laboratory tests showed severe hypoglycemia with a plasma glucose level of 36 mg/dL. The patient's HbA1c was 6.9%. Other initial blood tests and a head computed tomography (CT) scan revealed no significant abnormalities. His consciousness gradually recovered over several hours following glucose administration, leading to his admission for further investigation.

During hospitalization, EIAS was suspected due to the patient's history of glycemic instability coexisting with high insulin requirements. Although autoimmune diabetes could not be completely ruled out, the patient had a long‐standing diagnosis of type 2 diabetes, with stable glycemic control on both oral agents and insulin—an atypical course for autoimmune diabetes. Anti‐GAD antibody testing was also negative.

Based on these findings, his insulin regimen was changed to insulin aspart 8 units three times daily and insulin degludec 8 units in the evening, representing a significant reduction in the total daily dose. Following this change, his blood glucose control stabilized during the admission. Several days after admission, subsequent laboratory testing confirmed a high insulin antibody level of 8.7 U/mL (reference range: < 0.4 U/mL), which had been drawn upon presentation. The diagnosis of EIAS was made. The patient was discharged one week after admission. His glycemic control remained stable following discharge on the modified insulin regimen.

## 3 Discussion

This case report highlights two key aspects of EIAS observed in an elderly patient with diabetes. First, EIAS should be considered in the differential diagnosis for patients on insulin therapy who present with insulin resistance and glycemic instability, particularly severe hypoglycemia. Second, modifying or discontinuing the current insulin regimen is a crucial initial step for both diagnosis and management when EIAS is suspected. These observations underscore the diagnostic complexity of EIAS and demonstrate the therapeutic value of insulin regimen modification in affected individuals.

The paradoxical nature of EIAS, presenting with both severe hypoglycemia and features of insulin resistance (e.g., high insulin requirements like 74 units daily in our patient), often leads to diagnostic challenges. In this case, the insulin dose corresponded to approximately 1.06 units/kg/day, which exceeds the commonly accepted threshold of 1.0 units/kg/day used to define insulin resistance in patients with type 2 diabetes [4]. EIAS is frequently overlooked or misdiagnosed as symptoms mimic more common causes of hypoglycemia in insulin‐treated diabetics, such as insulin overdose, and a lack of high‐quality evidence hinders early identification [5]. Our case highlights this; initial tests for hypoglycemia did not include insulin and C‐peptide levels, as EIAS was not suspected. Although the insulin‐to‐C‐peptide ratio has been proposed as a diagnostic clue for EIAS [5, 6], caution is needed as exogenous insulin can lead to false‐positive results due to assay interference [5]. The subsequent high insulin antibody titer (8.7 U/mL) confirmed EIAS. The underlying pathophysiology involves antibodies binding exogenous insulin, creating a reservoir that unpredictably releases insulin, causing fluctuating glycemia [2]. Scatchard plot analysis, though unavailable, could further elucidate antibody properties [2].

The second key finding, the rapid stabilization of glycemic control following the switch to different insulin analogues at a markedly reduced total daily dose, demonstrates a primary management approach for EIAS. In our patient, glycemic stability was rapidly achieved by switching to different insulin analogues, which allowed for effective control at a markedly reduced total daily dose (32 units). Changing or stopping the current insulin is recognized as a core strategy [2, 5]. While successful in this case, some patients with severe EIAS may require additional interventions such as glucocorticoids or other immunosuppressive therapies to decrease antibody production or facilitate immune complex dissociation [2, 5]. Nevertheless, the ability to achieve stable glycemic control with a substantially lower total daily dose simply by switching insulin analogues in our patient highlights the potential efficacy of this initial approach and its diagnostic utility. It underscores that prompt empirical adjustment of the insulin regimen can be crucial for managing suspected EIAS and resolving dangerous glycemic instability.

## 4 Conclusion

EIAS represents a potentially overlooked cause of hypoglycemia in insulin‐treated individuals with diabetes. Clinicians should consider the possibility of EIAS in patients exhibiting both glycemic variability, such as nocturnal hypoglycemia, and apparent insulin resistance necessitating high insulin doses. When EIAS is suspected, modifying the therapeutic approach by changing or discontinuing the current insulin regimen is a crucial first step in both diagnosis and management.

## Author Contributions

**Kazuki Miyaue** contributed to the Conception, data acquisition, analysis, and drafting of the manuscript. **Hiroki Isono** supervised the clinical investigation and contributed to the final manuscript review. All authors read and approved the final version of the manuscript.

## Funding

The authors have nothing to report.

## Consent

This article is published with the written consent of the patient.

## Conflicts of Interest

The authors declare no conflicts of interest.

## Data Availability

Data sharing is not applicable to this article as no new data were generated or analyzed during the study.

## References

[1] P. E. Cryer , L. Axelrod , A. B. Grossman , et al., “Evaluation and Management of Adult Hypoglycemic Disorders: An Endocrine Society Clinical Practice Guideline,” Journal of Clinical Endocrinology and Metabolism 94, no. 3 (2009): 709–728. 10.1210/jc.2008-1410 19088155

[2] X. Hu and F. Chen , “Exogenous Insulin Antibody Syndrome (EIAS): A Clinical Syndrome Associated With Insulin Antibodies Induced by Exogenous Insulin in Diabetic Patients,” Endocrine Connections 7, no. 1 (2018): R47–R55. 10.1530/EC-17-0309 PMC5776673 29233817

[3] American Diabetes Association Professional Practice Committee , “6. Glycemic Goals and Hypoglycemia: Standards of Care in Diabetes – 2025,” Diabetes Care 48, no. Supplement_1 (2025): S128–S145. 10.2337/dc25-S006 PMC11635034 39651981

[4] T. J. Church and S. T. Haines , “Treatment Approach to Patients With Severe Insulin Resistance,” Clinical Diabetes 34, no. 2 (2016): 97–104. 10.2337/diaclin.34.2.97 PMC4833480 27092020

[5] H. Zhang , M. X. Yuan , and Q. Pan , “Insulin Autoimmune Syndrome: A Chinese Expert Consensus Statement,” Aging Medicine 8, no. 1 (2025): e70007. 10.1002/agm2.70007 PMC11845856 39990629

[6] T. Huynh , “Clinical and Laboratory Aspects of Insulin Autoantibody‐Mediated Glycaemic Dysregulation and Hyperinsulinaemic Hypoglycaemia: Insulin Autoimmune Syndrome and Exogenous Insulin Antibody Syndrome,” Clinical Biochemist Reviews 41, no. 3 (2020): 93–102. 10.33176/AACB-20-00008 PMC7731936 33343044

